# A computer-based avatar task designed to assess behavioral inhibition extends to behavioral avoidance but not cognitive avoidance

**DOI:** 10.7717/peerj.5330

**Published:** 2018-07-31

**Authors:** M. Todd Allen

**Affiliations:** School of Psychological Sciences, University of Northern Colorado, Greeley, CO, United States of America

**Keywords:** Avoidance, Virtual reality, Cognitive-behavioral avoidance scale, Computer based assessment, Personality assessment tools

## Abstract

Avoidance is a common feature of post-traumatic stress disorder (PTSD) as well as anxiety and depressive disorders. Avoidance can be expressed behaviorally as well as cognitively. Most personality assessments for avoidance involve self-report inventories which are susceptible to biased responding. The avatar task (Myers et al., 2016a) was developed as an objective measure of behavioral inhibition (BI) which is defined as a tendency for avoidance of unfamiliar people and situations. The avatar task has been demonstrated to screen avoidant behaviors related to BI, PTSD, as well as harm avoidance (HA) as measured by the Tridimensional Personality Questionnaire (TPQ). In the current work, the avatar task was tested with cognitive as well as behavioral avoidance as measured by the cognitive-behavioral avoidance scale (CBAS; Ottenbreit & Dobson, 2004). The CBAS includes four subscales which measure behavioral social (BS) avoidance, behavioral non-social (BN) avoidance, cognitive social (CS) avoidance, and cognitive non-social (CN) avoidance. It was hypothesized that avatar scores would be significantly positively related to behavioral, but not cognitive, avoidance. In addition, it was also hypothesized that performance on the avatar task would be more related to social than non-social behavioral avoidance. Participants completed the avatar task, the HA scale of the TPQ and the CBAS. Pearson’s product moment correlations revealed that avatar scores were significantly related to CBAS total scores as well as BS and BN scores, but not CS and CN scores. In addition, BS has a stronger relationship with avatar scores than BN avoidance which fits with the social aspects of the scenarios in the avatar task. A median split of the avatar scores produced a significant difference in scores on the behavioral but not the cognitive subscales. Overall, the current results supported the idea that the avatar task is measuring behavioral avoidance, specifically in social situations, rather than cognitive avoidance. Future work could adapt the avatar task to include scenarios similar to the cognitive items on the CBAS to create an objective measure of cognitive avoidance which may be relevant in measuring avoidance in depression and behavioral avoidance associated with PTSD as well as anxiety disorders.

## Introduction

Avoidance is a key symptom of post-traumatic stress disorder (PTSD) and anxiety disorders (American Psychiatric Association, 2013). In addition, avoidance is also related to depression (Ferster, 1973; Folkman & Lazarus, 1986; Kuyken & Brewin, 1994; Spurrell & McFarlane, 1995). Recent work has focused on avoidance-related personality temperaments such as behavioral inhibition (BI) or harm avoidance (HA) which are risk factors for the development of PTSD, anxiety disorders, and depression. BI is defined as a tendency for avoidance of unfamiliar people and situations (Kagan, Reznick & Snidman, 1987; Morgan, 2006). HA is defined as a tendency to avoid harmful stimuli including situations involving punishment, novelty, and non-reward (Cloninger, 1986; Cloninger, 1987). This work has examined a learning diathesis model of PTSD in which personality temperaments are associated changes in avoidance (e.g., Sheynin et al., 2014) and associative learning (e.g., Allen, Myers & Servatius, 2014) which may increase vulnerability to the development and maintenance of PTSD. As will be detailed, inhibited temperaments are related to the development of PTSD, anxiety disorders, and depression.

To satisfy the *DSM-5* criterion C for PTSD, an individual must persistently avoid either external reminders of trauma (i.e., behavioral avoidance) or trauma-related thoughts or feelings (i.e., cognitive avoidance), or both (American Psychiatric Association, 2013). While avoidance of traumatic or stressful situations is usually adaptive, avoidance can become maladaptive and disrupt normal life functioning. Avoidance behaviors have been identified as a significant predictor of PTSD (Charlton & Thompson, 1996; Marmar, 1996; Chang et al., 2003) as well as distinguishing between those at risk and not at risk for development of PTSD (North et al., 1999; Barlow, 2002; Karamustafalioglu et al., 2006; Marshall et al., 2006; O’Donnell et al., 2007). Avoidant tendencies (as measured by BI) are related to PTSD. Specifically, childhood BI is a risk factor for the later development of adult PTSD (North et al., 1999; Fincham et al., 2008; Kashdan, Morina & Priebe, 2009). BI also correlates with severity of PTSD symptoms (Myers et al., 2012; Myers, VanMeenen & Servatius, 2012; Servatius et al., 2017). In addition, Richman & Frueh (1997) reported high levels of HA in veterans with combat related PTSD. More specifically, high levels of HA are a risk factor for PTSD (Cloninger, Svrakic & Przybeck, 2006; Gil, 2005) while low levels are associated with resilience (Simeon et al., 2007).

Inhibited temperament is also linked to anxiety disorders. The relationship between PTSD and anxiety disorders is complicated in that PTSD is currently categorized as a trauma and stressor related disorder, but in the previous edition of the DSM (American Psychiatric Association, 2000) PTSD was categorized as an anxiety disorder. Avoidance of feared situations or stimuli is a symptom of anxiety disorders in the DSM-5 (American Psychiatric Association, 2013). BI has been identified as a vulnerability for the development of anxiety disorders such that childhood BI increases the risk for an individual to develop anxiety disorders in adulthood (Hirshfeld et al., 1992; Biederman et al., 1993; Svihra & Katzman, 2004; Pérez-Edgar et al., 2010) specifically, social inhibition (Kupper et al., 2011) and social anxiety disorder (Chronis-Tuscano et al., 2009; Lahat, Hong & Fox, 2011; Kupper & Denollet, 2014).

Avoidance has also been put forth as a symptom of depression. However, the relationship between avoidance and depression is not as well-established as that with PTSD and anxiety disorders. Leventhal (2008) proposed that depression comes about when maladaptive avoidance limits positive experiences. Following a negative event, a person may avoid similar situations to avoid further potentially negative events. By avoiding situations that have the potential for an undesirable outcome, the person misses out on the opportunity for possible desirable outcomes, and thus falls deeper into depression. This idea was supported by findings in which the relationship between avoidance and depression is mediated by reinforcement (Carvalho & Hopko, 2011), the relationship between negative life events and depression is moderated by cognitive avoidance in females, but not males, and the relationship between anxiety sensitivity and depression is mediated by experiential avoidance (Tull & Gratz, 2008). In addition, BI was found to lead to depression (Gladstone & Parker, 2006), but this relationship was moderated by social inhibition. HA has also been linked to depression (Brown et al., 1992; Svrakic, Przybeck & Cloninger, 1992; Strakowski et al., 1995; Chen et al., 2015). Jacobson & Newman (2014) conducted a study of the longitudinal development between anxiety and depression that revealed that avoidance is a mediator between early anxiety and depression over ten years later. Thus, avoidance and anxiety interact in the development of depression.

As can be seen from the literature reviewed, there is a strong overlap in regards to avoidance between PTSD, anxiety disorders, and depression. Specifically, the avoidance-related temperaments of BI and HA increase the risk of PTSD, anxiety disorders, and depression. One concern with measures of both BI and HA is that they are self-report paper and pencil questionnaires. BI is measured by the Adult and Retrospective Measures of Behavioural Inhibition (AMBI/RMBI; Gladstone & Parker, 2005). HA is measured with a subscale of the Tridimensional Personality Questionnaire (TPQ; Cloninger, Przybeck & Svrakic, 1991; Cloninger, Svrakic & Przybeck, 1993). However, the tendency to avoid inherent in both BI and HA may affect the accuracy of self-report. Some participants in military or veteran populations may exaggerate their responses towards non-avoidance related choices to avoid the diagnosis of PTSD. Emergency responders who develop PTSD may fear losing their jobs and thus avoid participation in research with PTSD questionnaires (Clohessy & Ehlers, 1999). In addition, some individuals may exaggerate responses towards avoidance related choices to receive a diagnosis of PTSD and increase disability benefits (Freeman, Powell & Kimbrell, 2008). Recent alternatives to paper and pencil self-report inventories assessing avoidance have included both behavioral avoidance tasks as well as computer-based assessments.

There has been some work on developing behavioral measures of avoidance in humans. Animal paradigms have utilized approach-avoidance paradigms in which approaching a reward (i.e., food or water) is in conflict with avoiding a punishment (i.e., an electric shock) as well as avoidance tasks in which a response (i.e., a lever press) to a warning signal avoids an impending aversive event (i.e., an electric shock). These animal paradigms have been adapted in studies of human avoidance which have employed computer-based avoidance tasks involving a loss of points or monetary reward or a negatively affective stimulus as the aversive event to be avoided (e.g., Aupperle et al., 2011; Sheynin et al., 2014). Specifically, a computer-based approach—avoidance task developed by Aupperle et al. (2011)—involved the participant moving an avatar along a runway between a positive and negative affective stimulus to indicate the participant’s preference for a specific outcome (either a reward of points or a punishment of a negatively affective picture). Approach behavior on the task was correlated with a self-reported tendency to approach reward and avoid punishment as measured by the Behavioral Inhibition/Activation Scale (BIS/BAS; Carver & White, 1994). This paradigm focused more on the conflict between approach and avoidance rather than avoidance per se and also did not involve a learning component.

A computer based avoidance learning task developed by Molet, Leconte & Rosas (2006) and adapted by Sheynin et al. (2014) does include a learning component. In this task, participants maneuver and spaceship to score points. A warning signal (a light) indicates an impending aversive event that results in points loss. Participants learn that they can avoid a points loss by hiding their spaceship in a safe zone in response to the warning signal. Importantly, subjects received no direct instructions, the warning signal or on how to escape from or avoid point loss. The vast majority of the participants learned the escape response with the aversive event, while most of them also learned to completely avoid point loss by performing an avoidance response to the warning signal (Sheynin et al., 2014). Enhanced avoidance responding was exhibited by HA individuals (Sheynin et al., 2014) and individuals self-reporting PTSD symptoms (Sheynin et al., 2017). Thus, this human avoidance task not only measures learning of an avoidance response, but also is sensitive to inhibited temperaments.

An alternative to using computer-based behavioral tasks to assess avoidance, is the use of a graphical representation of the user within an interactive virtual environment (i.e., an avatar) which has been put forth as a viable alternative to paper and pencil self-report questionnaires (Blascovich et al., 2002). The use of an avatar task to assess avoidance behaviors fits with an increased interest in the use of computer-based virtual reality in psychology and behavioral neuroscience for a variety of purposes including exposure therapy for anxiety disorders (for review see: Parsons & Rizzo, 2008), social interaction (Hoyt, Blascovich & Swinth, 2003), social skill training (Park et al., 2011), and sensation and perception (Waller, Beall & Loomis, 2004).

In addition, virtual reality tasks have been used for behavioral assessment of traumatic brain injury (TBI) or stroke patients as a substitute for paper and pencil inventories and simple motor tasks which have been criticized as not testing patients in a practical manner relevant to the real world (Lee et al., 2003). Avatar-based programs have also been used to assess aspects of schizophrenia including social anxiety (Park et al., 2009) and cognitive flexibility (Han, Kim & Kim, 2012). Thus, avatars and virtual reality have been successfully applied to a variety of psychological topic including assessment.

Continuing in this line of research using avatars or virtual reality for assessment, Myers et al. (2016a) developed a computer-based task avatar task as an alternative to paper and pencil self-report based inventories assessing avoidance related personality. In this avatar task, participants selected and guided an avatar through two scenarios in which they indicate how they would respond in real life when interacting with strangers. The scenarios in the avatar task were based on the items in the AMBI (Gladstone & Parker, 2005).

Performance on the avatar task predicted scores on the AMBI in a sample of undergraduates. The relationship between scores on the avatar task and the AMBI was stronger when individuals were instructed to act as they would in real life as compared to when instructed to act like a typical university student. Allen, Jameson & Myers (2017) reported that scores on the avatar task also had a strong relationship with another measure of inhibited temperament, HA as measured with the TPQ in an undergraduate sample. The avatar task has also been tested in a sample of veterans self-reporting PTSD symptoms on the PTSD Checklist (PCL; Blanchard et al., 1996). Myers et al. (2016b) found that scores on the avatar task were strongly related to avoidance symptoms (i.e., criterion 3 of the DSM-5). Overall these limited studies have provided evidence that the avatar task measures some aspect of avoidance related to inhibited temperaments and PTSD, but one issue concerning the avatar task, much like self-report inventories, is whether it might not measure distinct concepts, but rather, a higher order construct (Suls & Bunde, 2005; Ketterer et al., 2002). The ability of the avatar task to assess BI, HA and PTSD symptoms may reflect an overall avoidance construct rather than individual distinct concepts.

In an attempt to determine if the avatar task assesses distinct concepts of avoidance, the current study tested the avatar task with cognitive avoidance, as well as behavioral avoidance. Behavioral avoidance involves escaping or avoiding a stressful situation while cognitive avoidance involves attempts to avoid or escape thinking about traumatic situations or problems (Ottenbreit & Dobson, 2004). Cognitive avoidance can take a range of forms, including intentional attempts at thought/memory suppression, efforts to dissociate from the emotions related to the traumatic experience, and engaging in rumination (Ehlers & Clark, 2000). A paper and pencil measure that assesses both behavioral and cognitive avoidance is the cognitive behavioral avoidance scale (CBAS; Ottenbreit & Dobson, 2004). The CBAS is a 31 item self-report measure of avoidance consisting of four subscales: Behavioral social (BS), behavioral non-social (BN), cognitive social (CS), and cognitive non-social (CN) that are summed to a total CBAS score. Ottenbreit & Dobson (2004) suggested that the use of self-report measures are a limitation and called for the development of a behavioral avoidance task that would be related to real life avoidance strategies. The avatar task is an attempt in the development of a behavioral avoidance task that incorporates real life scenarios that can be compared to performance on the CBAS.

In the current study, the author explored the relationship between the CBAS and the avatar task. The current study utilized in a convenience sample of undergraduates which corresponds to the development of the CBAS (Ottenbreit & Dobson, 2004) and the avatar task (Myers et al., 2016a) with undergraduate samples which were later extended to clinical samples (Ottenbreit, Dobson & Quigley, 2014; Myers et al., 2016b). The author also focused on HA in the current study based on the combined findings of a significant positive relationship between CBAS total and subscales scores and HA (Ottenbreit & Dobson, 2004) and a positive relationship between HA and scores on the avatar task (Allen, Jameson & Myers, 2017). The first goal of the current study was to replicate the prior findings of a significant relationship between HA and the CBAS. Based on positive relationship between CBAS and HA scores it was hypothesized that HA would be strongly correlated to the CBAS total score and subscale scores. The second goal of the current study was to evaluate how avatar scores are related to the CBAS total score and the four subscales. Based on the positive relationship between scores on the avatar task and HA scores (Allen, Jameson & Myers, 2017), it was hypothesized that performance on the avatar task would be related to avoidance as measured by the total CBAS score. More specifically, it was also hypothesized that scores on the avatar task would be related to behavioral avoidance subscales, but not cognitive avoidance subscales. This hypothesis came from the fact that the decision points in the scenarios in the avatar task were designed to parallel items on the AMBI which assesses behavioral inhibition. The avatar task also asks what a participant would do in particular social situation, not what the participant is thinking. Therefore, it was not designed to assess cognitive avoidance. In addition, it was hypothesized that the avatar task would have a stronger relationship with the social behavior scale than the non-social behavioral scale based on the novel social situations inherent in the party and volunteering scenarios in the avatar task.

The third goal of the current study was to assess the ability of the avatar task to differentiate to performance on the CBAS. Previously, Allen, Jameson & Myers (2017) reported that HA individuals (as defined by a standard cut off score) had significantly higher avatar scores than non-HA individuals. Specifically, it was hypothesized that individuals with high avatar scores should score high on the behavioral subscales, but not the cognitive avoidance scales.

## Methods

### Participants

One hundred and six undergraduates (80 females and 26 males) from an introductory psychology course voluntarily participated in the study as part of a research requirement. The participants had a mean age of 19.2 years (SD = 2.7, range 18–36 years) and a mean education level of 12.9 years (SD = 1.3). The ethnicity of our sample was mainly Caucasian (*n* = 58), followed by Hispanic (*n* = 18), African-American (*n* = 6), East Asian (*n* = 4), South Asian, (*n* = 2), multi-racial (*n* = 15), and other (*n* = 2). All participants read and signed an informed consent form before participating. All procedures were approved by the Institutional Review Board at University of Northern Colorado (approval number 799609-4).

### Instruments

Demographic information was collected including gender, age, years of education, and race/ethnicity through a brief questionnaire. Participants also completed the Tridimensional Personality Questionnaire (TPQ; Cloninger, Przybeck & Svrakic, 1991). The TPQ consists of 100 true/false iems assessing how the individual feels or behaves in various daily situations. The HA scale consists of 33 items (Cloninger, 1986; Cloninger, 1987). Individuals scoring 12 or higher on the HA scale are categorized as harm avoidant or “HA” and the remainder as non-avoidant or “non HA” based on the methodology of Cloninger, Przybeck & Svrakic (1991).

Participants also completed the CBAS (Ottenbreit & Dobson, 2004) which is a 31-item self-report measure of avoidance. The CBAS consists of four subscales of behavioral social (BS) avoidance (“I find that I often want to leave social gatherings.”), behavioral non-social (BN) avoidance (“I find myself avoiding tasks and assignments that are really important.”), cognitive social (CS) avoidance (“When I experience confusion in my relationships, I do not try to figure things out.”), and cognitive non-social (CN) avoidance (“I distract myself when I start to think about my work/school performance.”). Participants use a 5-point scale ranging from “not at all true for me” to “extremely true for me” to respond to each item. Scores on the four subscales are summed to create the CBAS total score which can range from 31 to 155 with higher scores indicating more avoidance.

### Computer-based task

**Figure 1:**
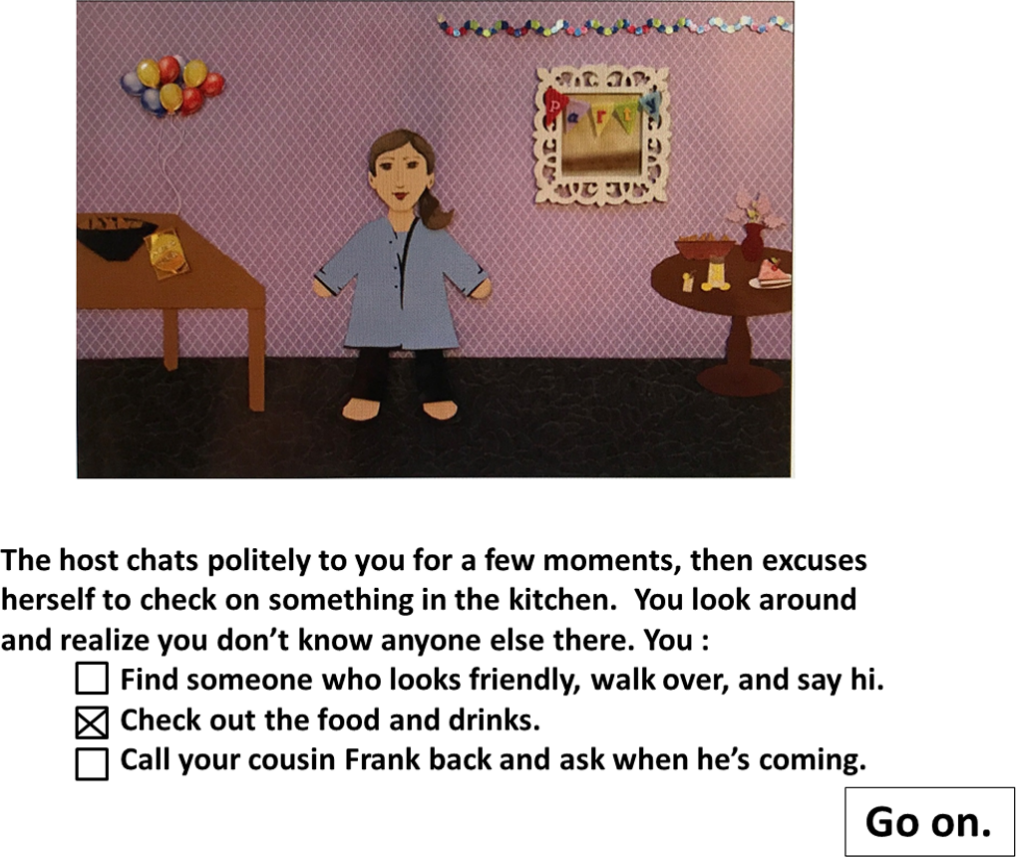
Sample screen capture from the computer-based avatar task. In the first scenario, the avatar was invited to a party where she did not know anyone there except her cousin. At this choice point the avatar has a choice between a non-inhibited responses, an intermediate response, and an inhibited response. In this case, the intermediate response was chosen.

The computer-based task has been described in previous reports (Myers et al., 2016a; Myers et al., 2016b; Allen, Jameson & Myers, 2017). The task took about 10 min for participants to complete. On the first screen, the participant saw a selection of four male and four female avatars with a range of skin and hair colors from which he/she could choose from to represent him/her in the task. The task itself consisted of one scenario in which the avatar was attending a party full of strangers and a second scenario in which the avatar was volunteering at a charity building project. The scenarios included twenty decision points with a short text description of an event and an image showing the avatar experiencing this event. Each decision point included response options between a relatively avoidant, relatively non-avoidant, and neutral behavior (Fig. 1). All participants experienced the same sequence of events and response options regardless of their responses. At each choice point, the participant received 2 points for selecting the avoidant option, 1 for the neutral option, and 0 for the non-avoidant option. Total scores could range from 0 to 40 with higher scores indicating higher levels of avoidance. Total scores were not reported to the participants. The entire study was completed within 30 min.

### Data analysis

Pearson’s product moment correlations were calculated between total scores on the avatar task and the scores from HA subscale of the TPQ, the CBAS total score as well as the four CBAS subscales. A Bonferroni correction was used to protect against inflated risk of family-wise type-I error for multiple correlations. This correction resulted in the significance level for the Pearson’s correlation being reduced to .05∕6 = 0.0083. Significant differences between Pearson’s correlations were calculated with a Fisher r to z transformation. A median split of the avatar scores resulted in high and low avatar groups which were compared with a *t*-test.

## Results

Computer failure resulted in loss of data for nine participants. The final sample for analysis consisted of ninety eight individuals (74 females and 24 males) with an average age of 19.3 years (SD = 2.8).

### Paper and pencil inventories

Table 1 shows the mean HA scale and CBAS total scores including scores for the four CBAS subscales for all participants with separate listings for females and males. There were no significant gender differences on the HA, CBAS total score and CBAS subscales. However, there was a non-significant trend that males had higher scores on the HA and CS scales than females.

**Table 1:** Scores on the HA and CBAS inventories.

	Total Mean (SD)	Female Mean (SD)	Male Mean (SD)	Gender effect significance level
Harm avoidance score	15.3 (8.2)	15.6 (7.3)	10.6 (6.5)	*p* = 0.09
CBAS total score	60.6 (18.7)	59.4 (17.7)	63.6 (21.1)	*p* = 0.39
Behavioral social avoidance	16.8 (7.1)	16.9 (7.1)	16.1 (7.1)	*p* = 0.66
Behavioral non-social avoidance	13.1 (4.0)	12.9 (3.7)	13.5 (5.0)	*p* = 0.57
Cognitive social avoidance	13.5 (5.3)	13.0 (5.1)	15.1 (5.5)	*p* = 0.09
Cognitive non-social avoidance	17.2 (6.3)	16.8 (6.4)	18.9 (6.0)	*p* = 0.14

As shown in Table 2, the CBAS total score and subscales were all significantly related (all *r*’s >0.80). These findings are similar to the levels of internal consistency reported by Ottenbreit & Dobson (2004) for an undergraduate sample and by Ottenbreit, Dobson & Quigley (2014) for a sample of clinically depressed women. HA also had a significant positive relationship to the total CBAS score as well as the BS, BN, and CN subscales.

**Table 2:** Correlation Matrix for the HA subscale scores and CBAS total and subscale scores.

Measure	1	2	3	4	5	6
1. Harm avoidance	–					
2. CBAS	**0.59**	–				
3. Behavioral social avoidance	**0.66**	**0.82**	–			
4. Behavioral non-social avoidance	**0.57**	**0.80**	**0.61**	–		
5. Cognitive social avoidance	0.22	**0.81**	**0.50**	**0.48**	–	
6. Cognitive non-social avoidance	**0.40**	**0.85**	**0.49**	**0.63**	**0.69**	–

### Responses on the AVATAR

**Figure 2:**
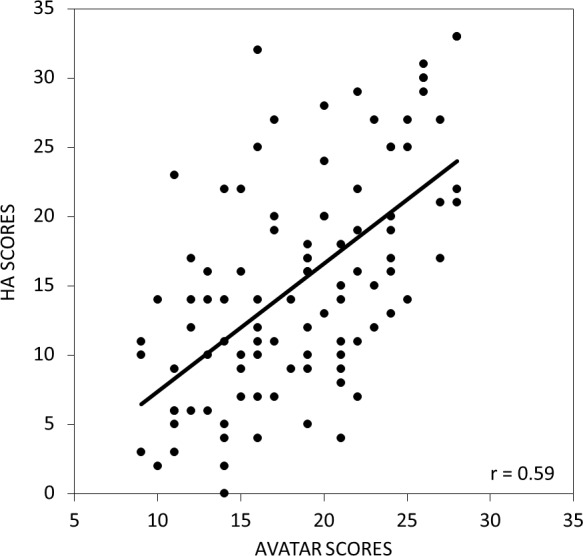
The relationship between the scores on avatar task and the harm avoidance (HA) subscale of the Tridimensional Personality Questionnaire. Total scores on the avatar task were strongly positively correlated with the HA subscale scores from the Tridimensional Personality Questionnaire.

The mean avatar score was 18.4 (SD = 5.3). While females (mean = 18.8, SD = 5.4) tended to have higher avatar scores than males (mean = 17.1, SD = 4.5) this difference was not significant (*p* = 0.12). As shown in Fig. 2, there was a significant positive relationship between the total AVATAR score and the HA subscale of the TPQ (*r* = 0.59, *p* < 0.001).

**Figure 3:**
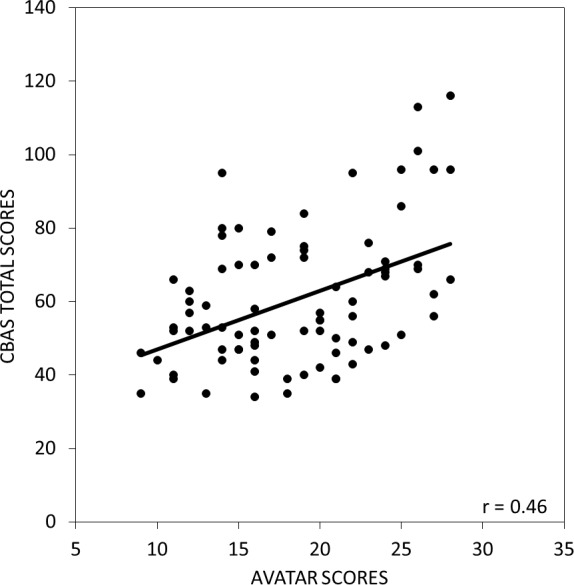
The relationship between scores on the avatar task and the CBAS total score. Total scores on the avatar task were strongly positively correlated with the total CBAS scores.

**Figure 4:**
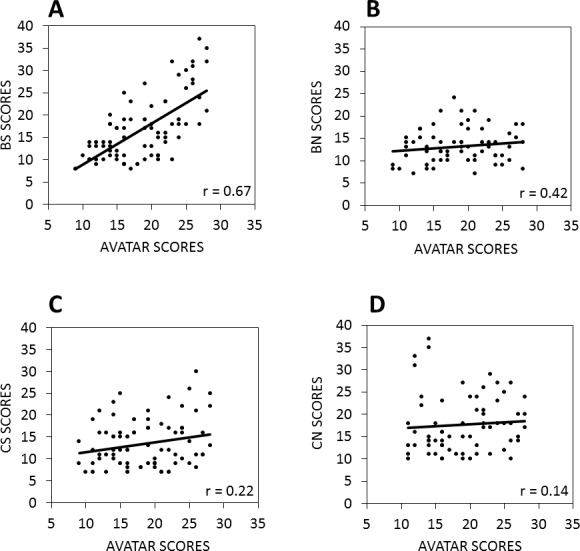
The relationship between scores on the avatar task and the CBAS subscales. Total scores on the avatar task were strongly positively correlated with (A) the behavioral social (BS) subscale and (B) the behavioral non-social (BN) subscale, but not (C) the cognitive social (CS) and (D) cognitive non-social (CN) subscales of the CBAS.

There was also a strong positive relationship between scores on the avatar task and the total CBAS score (*r* = 0.48, *p* < 0.001) as shown in Fig. 3. In addition, while avatar scores were significantly related to both the BS (*r* = 0.70, *p* < 0.001) and the BN (*r* = 0.47, *p* < 0.001) subscale scores as shown in Figs. 4A and 4B, the BS scores had a stronger relationship with the avatar scores than the BN scores (*z* = 2.4, *p* < 0.05). There were no significant relationships between scores on the avatar task and the Cognitive Social subscale (*r* = 0.26, *p* = .010) and between the avatar scores and the Cognitive Non-social subscale (*r* = 0.20, *p* = .046) as shown in Figs. 4C and 4D.

**Figure 5:**
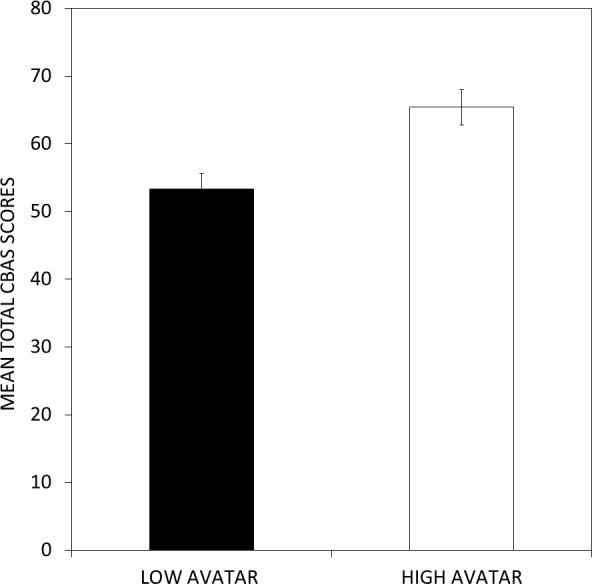
Differences in CBAS total scores as a function of scores on the avatar task. A median split of the avatar scores can be used to differentiate the CBAS total score. Individuals with high scores on the avatar task exhibited significantly higher CBAS total scores than individuals with low scores on the avatar task.

**Figure 6:**
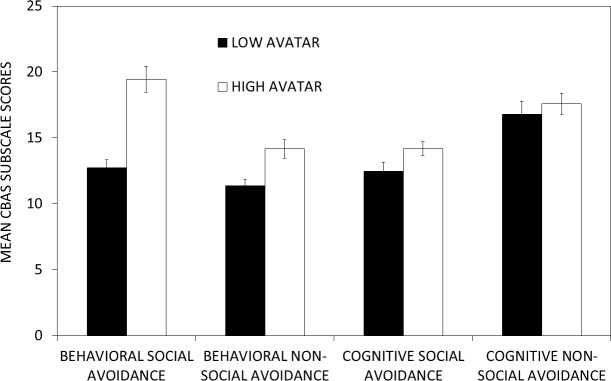
Differences in the CBAS subscale scores as a function of scores on the avatar task. A median split of the avatar scores can be used to differentiate performance on the behavioral social (BS) and behavioral non-social (BN) avoidance subscales, but not the cognitive social (CS) and cognitive non-social (CN) avoidance subscales. Individuals with high scores on the avatar task exhibited significantly higher BS and BN scores than individuals with low scores on the avatar task. There were no significant differences in CS and CN scores for individuals with high and low avatar scores.

Using a median split of the avatar scores at a score of 18.5 resulted in two groups such that the high avatar group had significantly higher mean CBAS total scores than the low avatar group (*t*(46) =  − 3.459, *p* < 0.001) as shown in Fig. 5. This median split of the avatar scores was also used to analyze differences in the CBAS subscale scores. The high avatar group had significantly higher scores than the low avatar group on the BS and BN scales (both *p* values <0.005), but not on the CS and CN scales (*p* = 0.19 and *p* = 0.38, respectively) as shown in Fig. 6.

## Discussion

The current study investigated how a computer-based avatar task designed to assess behavioral inhibition would relate to cognitive as well as behavioral avoidance as measured by the CBAS. Previously, the avatar task was found to be related to PTSD cluster C (avoidance) symptoms (Myers et al., 2016b) as well as HA avoidance (Allen, Jameson & Myers, 2017).

The first goal of the study was to replicate the findings of Ottenbreit & Dobson (2004) for the relationship between harm avoidance (HA) and CBAS total scores. The current finding of a strong relationship between HA and the CBAS total score as well as varying relationships with the four subscale scores fits with the previous report of Ottenbreit, Dobson & Quigley (2014) in which there were correlations of 0.50 or higher between HA and the CBAS total score and the BS and BN scores while the correlations between HA and the CS and CN scores were between 0.32 and 0.38.

Based on females having higher rates of anxiety disorders than males, gender effects were examined for the HA and CBAS scores. In the current study, females tended to have higher HA than males, but this difference only approached significance. This finding is in contrast to findings of higher HA in females in a meta-analysis of thirty two studies (Miettunen et al., 2007) as well as in a previous study with the avatar task (Allen, Jameson & Myers, 2017). There were also no significant differences between males and females for the CBAS total score or any of the subscales in the current study. However, there was a non-significant trend where males tended to have higher levels of cognitive social avoidance than females. Ottenbreit & Dobson (2004) found males had higher total CBAS scores as well as higher scores on the BS, CS, CN subscales but not the BN subscales as compared to females. However, subsequent studies have reported no gender effect on CBAS total scores (Moulds et al., 2007; Carvalho & Hopko, 2011). Overall, the current non-significant gender effects for CBAS and HA scores are in the same direction as some previous reports. However, the non-significant gender effects in the current study may be due in part to the small sample of males (*n* = 24) which may have lacked the power to detect gender differences. Future work with the avatar task and CBAS should include more males to further explore possible gender differences.

The second goal of the current was to investigate the relationship between the avatar task and the CBAS. The mean avatar score (18.5) was close to the range of scores reported for the avatar task for samples of undergraduates (Myers et al., 2016a; Allen, Jameson & Myers, 2017) but lower than those reported for PTSD patients (Myers et al., 2016b). This lack of a significant gender effect for the avatar task was consistent with prior reports (Myers et al., 2016a; Myers et al., 2016b; Allen, Jameson & Myers, 2017).

As hypothesized, the avatar task was significantly related to the total CBAS score. This finding fits with previous reports that the avatar task is related to BI (Myers et al., 2016a), as well as harm avoidance (Allen, Jameson & Myers, 2017), as well as avoidance symptoms of PTSD (Myers et al., 2016b). The current work sought to further refine the relationship of the avatar task to different types of avoidance, specifically behavioral and cognitive avoidance as measured by the CBAS. Scores on the avatar task had a significant positive relationship to the BS and BN scores, but not to the CS and CN scores. This finding fits with the idea that the avatar task was designed to assess behavioral inhibition rather than cognitive avoidance. The scenarios in the avatar task asked about what the participant would do, not what he/she thinks.

Another finding from the current study was that the avatar task had a stronger relationship to behavioral social avoidance than behavioral non-social avoidance. This finding also fits with the idea that the scenarios in the avatar task involve social interactions with strangers (i.e., a party and volunteering at a construction site). In addition, BI has been linked to social inhibition (SI) in that SI in adulthood may very well be preceded by BI in childhood (Kupper et al., 2011).

Based on the relationship between the avatar task and HA as well as the relationship between CBAS and HA, the author included this measure of avoidance in the current analysis. The mean HA score (15.3) was higher than recently reported (mean = 12.1, Sheynin et al., 2014; mean = 12.8, Sheynin et al., 2014; mean = 12.4, Allen, Jameson & Myers, 2017). The current study replicated the finding of Allen, Jameson & Myers (2017) that HA was related to the avatar task. The current study found a stronger relationship between HA and the avatar task than previously reported (Allen, Jameson & Myers, 2017). Previously HA relationship was at a level of *r* = 0.42 while the current finding was *r* = 0.59.

The third goal of the current study was to investigate the ability of the avatar task to differentiate behavioral and cognitive avoidance. To be relevant for diagnosis, it is not enough that scores on the avatar task are simply related to measures of avoidance, but that the avatar scores can be used to differentiate individual’s scores. A median split of the avatar scores at a score of 16.5 was used to group individuals as high and low avatar scores. Individuals who scored high on the avatar task had significantly higher scores on the CBAS task as well and the BS and BN subscales, but not the CS and CN subscales. The overall findings supported our hypothesis that performance on the avatar task differentiated behavioral avoidance but not cognitive avoidance also fit with the strength of relationships found between the avatar task and the CBAS subscales.

The differences in the relationships between performance on the avatar task and the behavioral and cognitive avoidance subscales may signify the difference between the relationship between the avatar task and PTSD and anxiety disorders as compared to depression. The CBAS was developed in the context of avoidance and depression rather than avoidance and anxiety disorders. Avoidance has been put forth as the mechanism through which sadness becomes depression (Leventhal, 2008). Ottenbreit & Dobson (2004) found that cognitive-behavioral avoidance was related to both anxiety disorders and depression and that the relationship between avoidance and depression approached the strength of the relationship between avoidance and anxiety disorders. Blalock & Joiner (2000) reported that cognitive avoidance coping strategies moderate the relationship between negative life events and depression in females, but not males. Experiential avoidance was associated with symptoms of depression when controlling for PTSD symptoms (Tull et al., 2004). Dickson, Ciesla & Reilly (2012) reported that cognitive avoidance was a robust predictor of rumination, worry, sadness, and anxiety while behavioral avoidance was only predictive of anxiety. Ottenbreit & Dobson (2004) reported that while higher levels of both behavioral and cognitive avoidance were related higher levels of depression, cognitive non-social avoidance had strongest relationship to depression. The current findings support the idea that the avatar task may be more relevant for measuring behavioral avoidance as it relates to anxiety more so than cognitive avoidance as it relates to depression.

There are several limitations concerning the sample of the current study which can be used in designing future work. As previously discussed, a limited number of males may have limited the ability to identify possible gender effects. Another limitation is the ethnic makeup of the current sample. There was a lack of ethnic diversity in that over half of our sample was Caucasian. Previous studies have reported that Asians used avoidant strategies more so than Caucasians (Bjorck et al., 2001; Ottenbreit & Dobson, 2004). Cross cultural explorations of PTSD, anxiety disorders, and depression could be undertaken with the avatar task to further explore possible ethnic differences in avoidance with these disorders.

The current study also utilized undergraduates with no regard for clinical diagnosis of PTSD, anxiety disorders or depressive disorders. The current study parallels initial work with the CBAS (Ottenbreit & Dobson, 2004) and the avatar task (Myers et al., 2016a) that was limited to non-clinical samples. Future work can attempt to replicate the current findings with clinically depressed or anxious individuals. Just as the original finding of a relationship between scores on the avatar task and BI reported by Myers et al. (2016a) was extended in a study in PTSD patients (Myers et al., 2016b), the current study with CBAS should be replicated and extended with clinical samples. In addition the avatar task may be valuable as an assessment tool for individual’s co-morbidity of PTSD and depression.

Another limitation of the current study is the time limit of 30 min to complete the avatar task as well as the paper and pencil inventories. Based on this time limit, the current study did not include the AMBI which has been consistently found to be related to the avatar or other measures of anxiety. The current study focused on the topic of the behavioral and cognitive avoidance (measured with the CBAS) and did not extend to other measures of avoidance or anxiety.

## Conclusion and Future Work

Future work can also compare results on the avatar task to avoidance learning tasks which have been found to be related to BI and HA. The AMBI and HA scale of the TPQ have been used to categorize individuals as avoidant prone who exhibited enhanced classical eyeblink conditioning (Holloway et al., 2014; Allen, Myers & Servatius, 2014; Allen, Jameson & Myers, 2017) and as well as enhanced avoidance in computer-based tasks (Sheynin et al., 2014). These studies should be repeated using the avatar task as a substitute for paper-and-pencil inventories such as the AMBI and TPQ to determine if avatar scores can predict performance on these learning tasks. The avatar task can also be tested with other measures of anxiety to determine how much it generalizes from behavioral avoidance. The use of the avatar task as a predictor of performance on avoidance tasks would answer the call by Ottenbreit & Dobson (2004) for behavioral avoidance tasks that can be related to real world scenarios like those in the avatar task.

Overall, the current study lends support for the Myers et al. (2016a) avatar task being an objective measure of behavioral avoidance, but not cognitive avoidance. The avatar task was also more related to behavioral social avoidance more so than behavioral non-social avoidance. Future work can also attempt to adapt the avatar task from behavioral avoidance to include cognitive avoidance by using items from the CBAS. Scenarios could be designed in which participants are asked explicitly about their thoughts rather than their actions. The current study helps to refine the extent to which this computer-based task relates to various aspects of avoidance. The ability of the avatar task to positively correlate with behavioral, but not cognitive avoidance lends support to continued work with the avatar task as a viable substitute for paper and pencil measures of avoidance as related to PTSD, anxiety disorders, and depression.

##  Supplemental Information

10.7717/peerj.5330/supp-1 Supplemental Information 1 Raw scores on the HA, CBAS inventories and avatar task

## Additional Information and Declarations

### Data Availability

The following information was supplied regarding data availability: The raw data are provided in Supplemental File.
